# Supplementary material for: Probing RNA structural landscapes across Candida yeast genomes
Source: Front Microbiol. 2024 Feb 26;15:1362067. doi: 10.3389/fmicb.2024.1362067 (PMC10926079; doi:10.3389/fmicb.2024.1362067)
Supplement: Supplementary file 1 [file Data_Sheet_1.pdf]

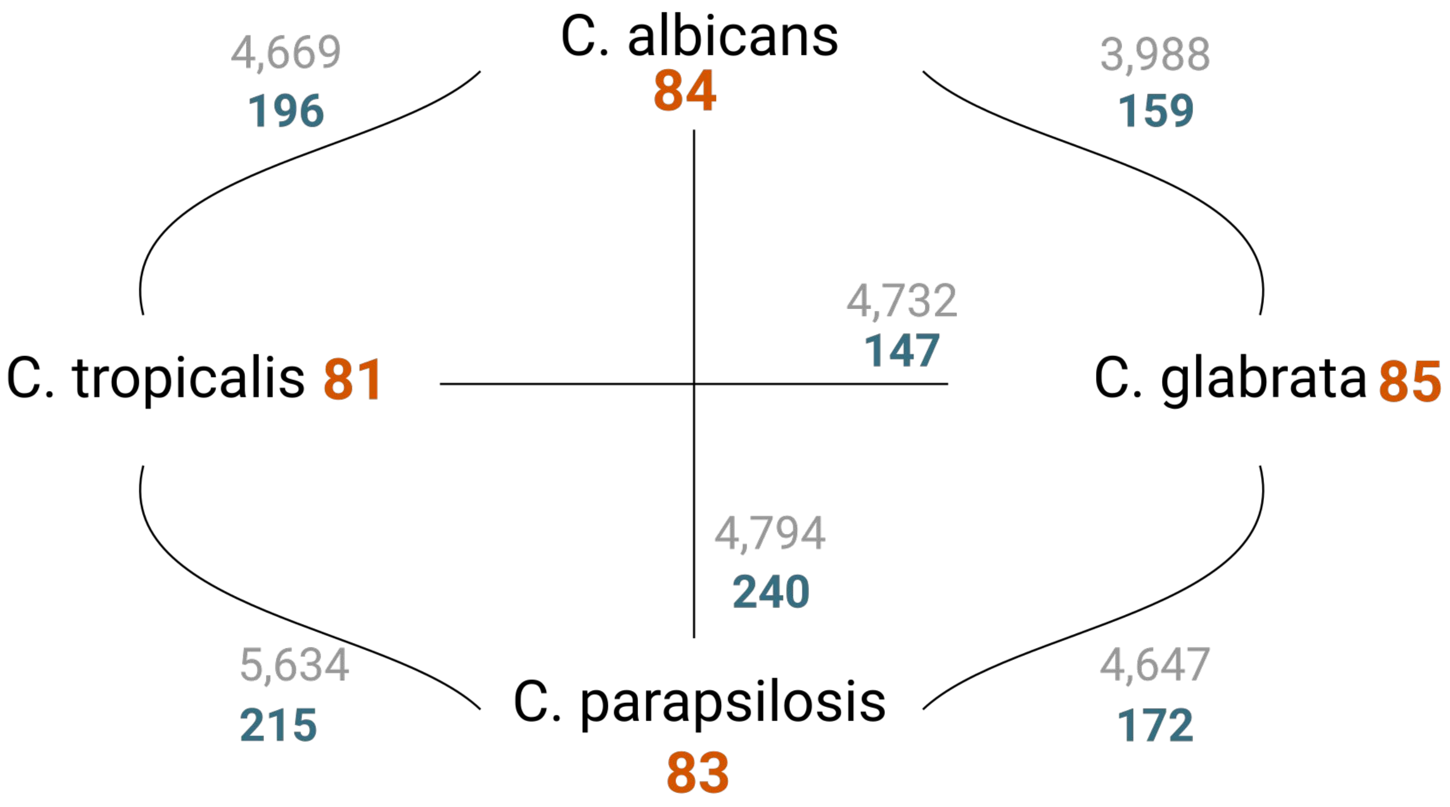

Figure S1. Genes with orthology information in the 4 *Candida* species. The numbers in blue indicate the number of genes with good nextPARS data, and the numbers in red represent those with good nextPARS data that are detected as orthologs in the pairs of species.

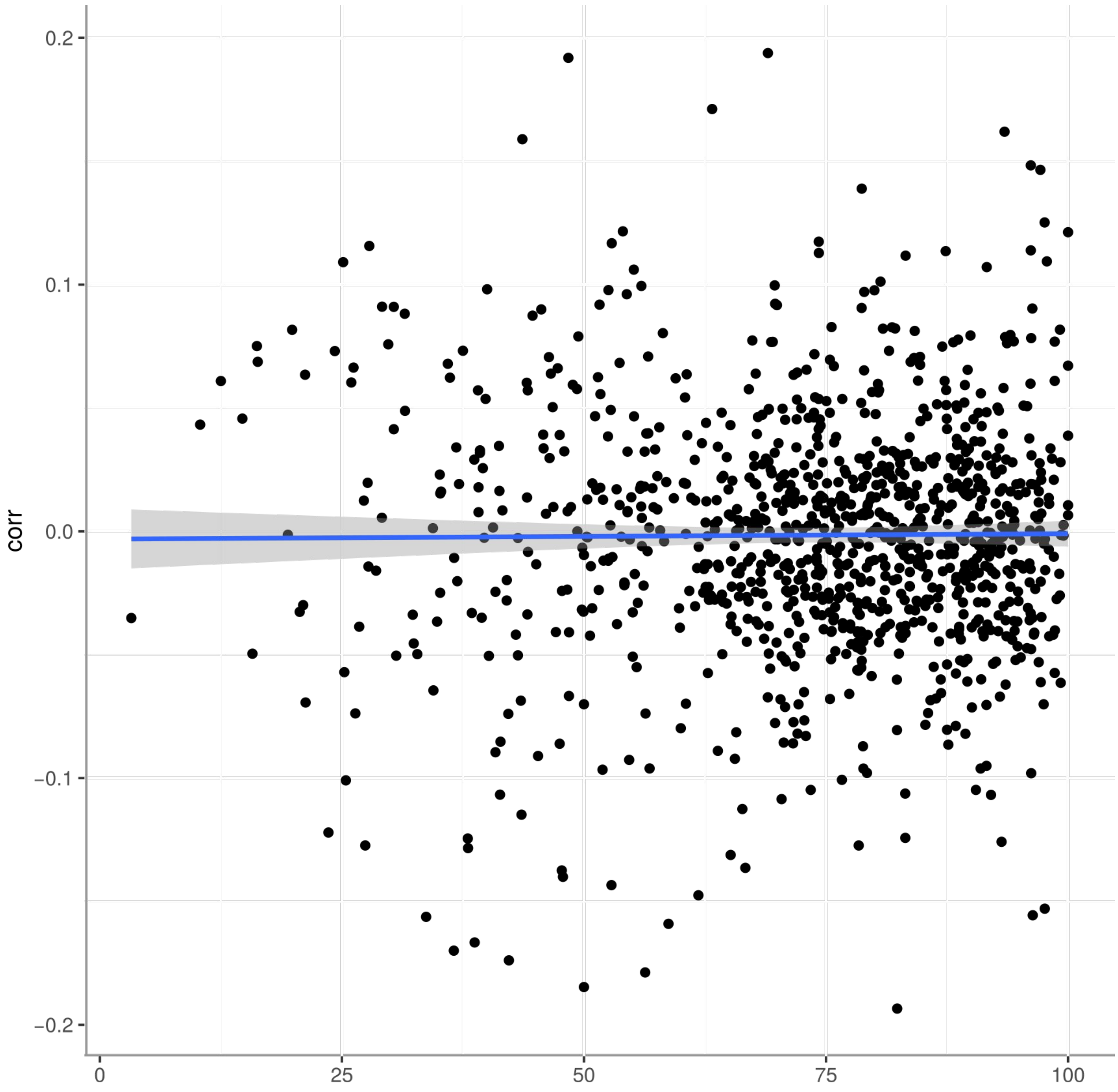

Figure S2. Correlations between shuffled scores. Dots indicate each pair of orthologs where we shuffled the positions of nextPARS scores and their alignments retrieved from different species.

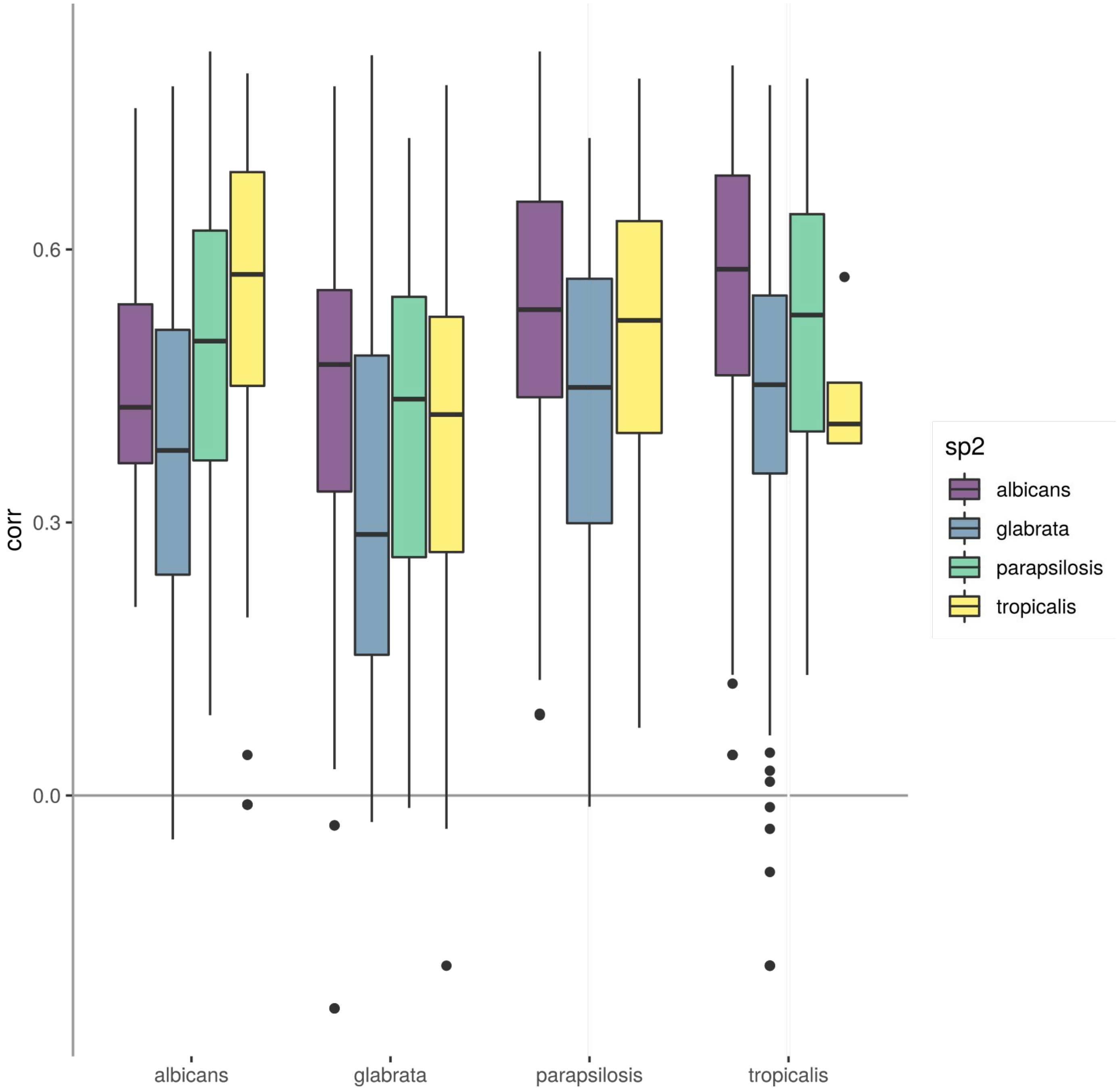

Figure S3. Correlation between sequence and structure in orthologs for nextPARS score in aligned positions

A B

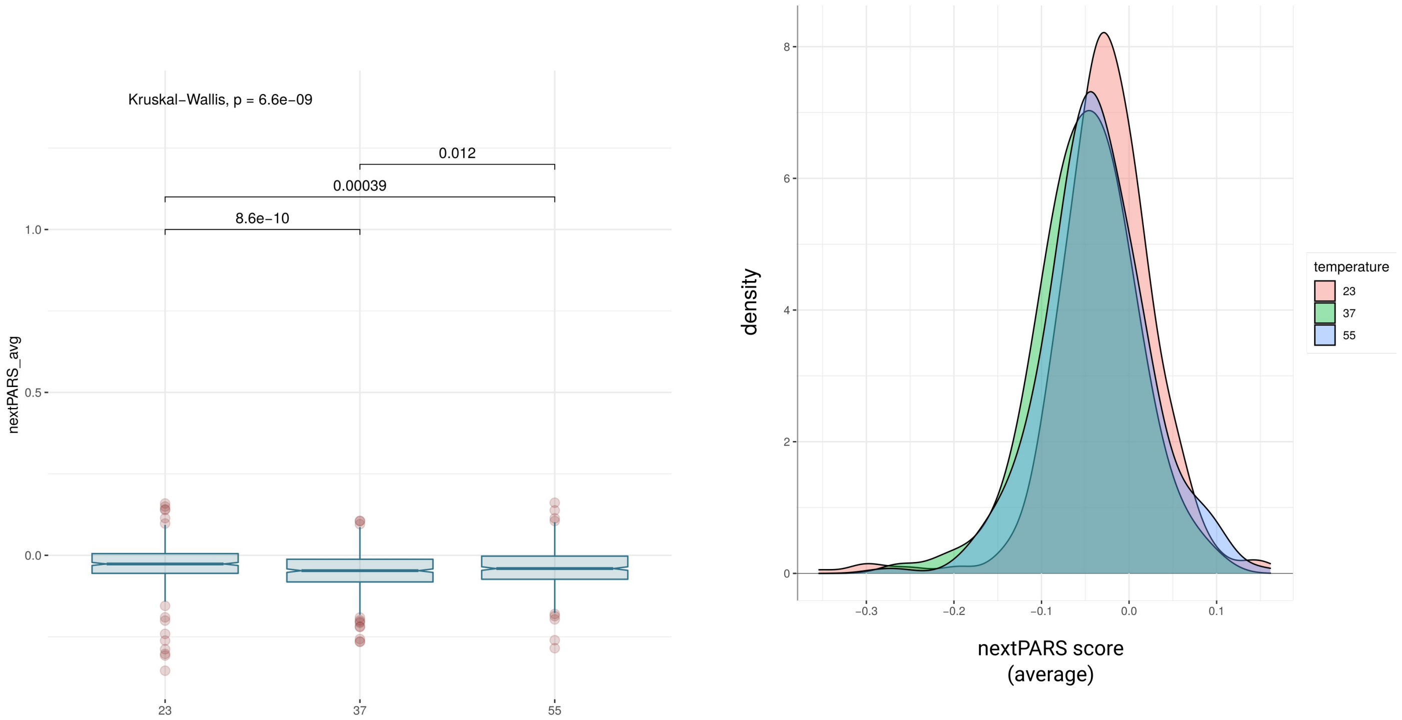

Figure S4. C Parapsilosis at different temperatures. **(A)** Box plot of nextPARS score for C. parapsilosis mRNAs at different temperatures. **(B)** Density plot of nextPARS score comparing conserved positions against non-conserved ones at three different temperatures.

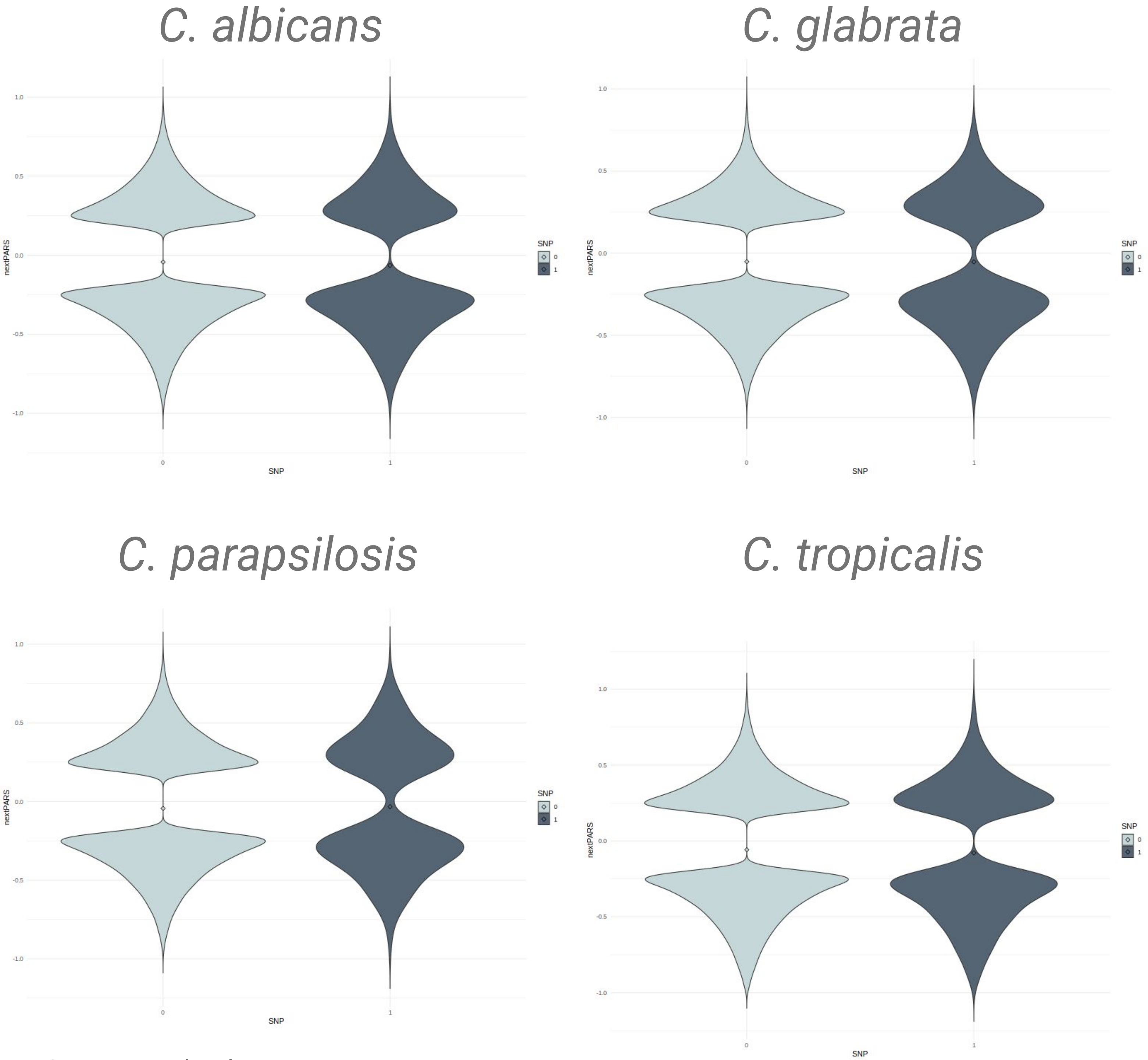

Figure S5. Violin plot comparing nextPARS scores in genomic loci with single nucleotide polymorphisms (SNPs) to those without reported SNPs across four *Candida* species.

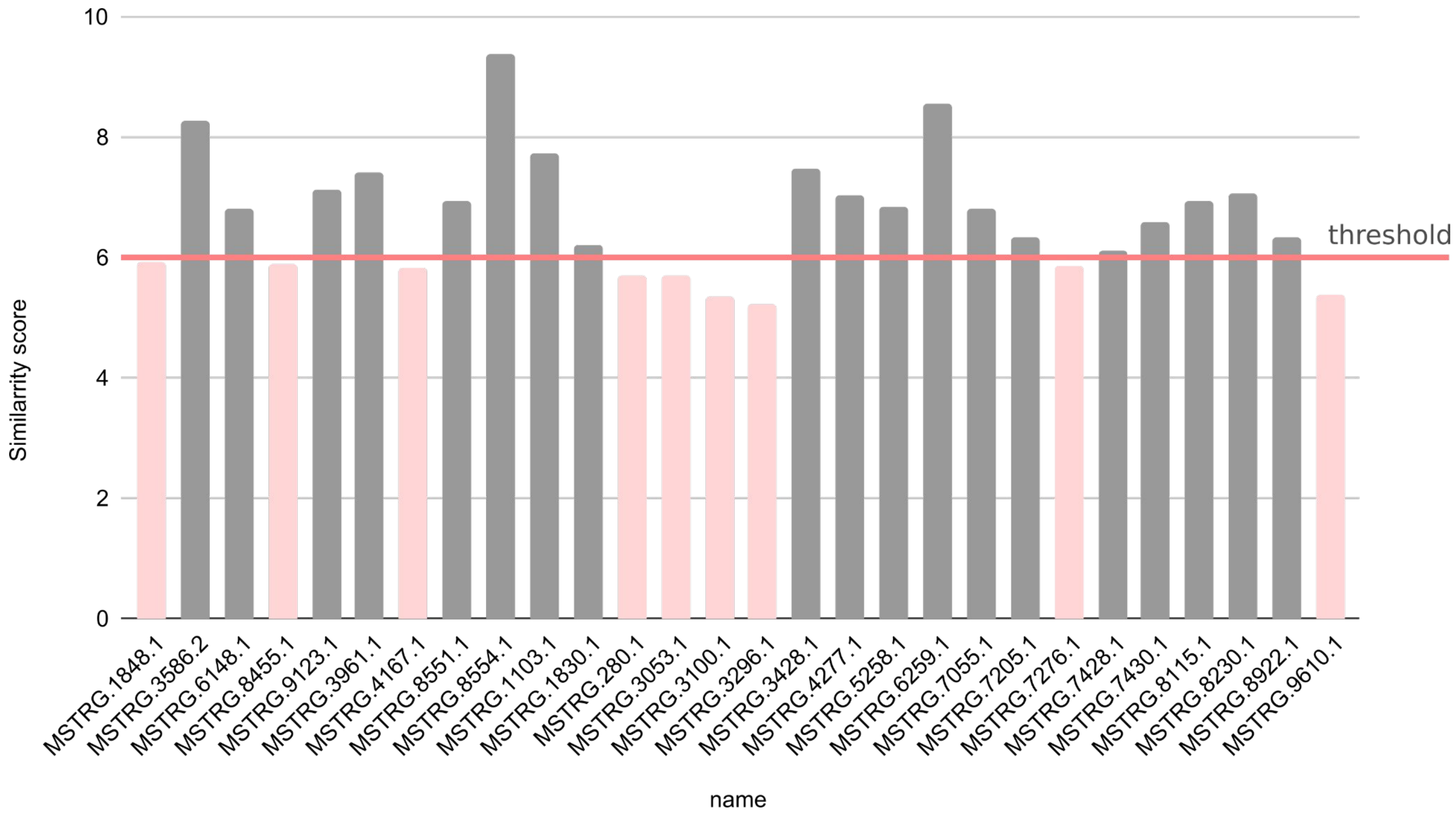

Figure S6. Bar plot illustrating the similarity scores for various long non-coding RNAs (lncRNAs), comparing the scores obtained with experimental constraints (nextPARS data) to predictions made without these constraints. The threshold for significance (similarity score below 6) is indicated by a red line, with lncRNAs exceeding this threshold depicted in grey.

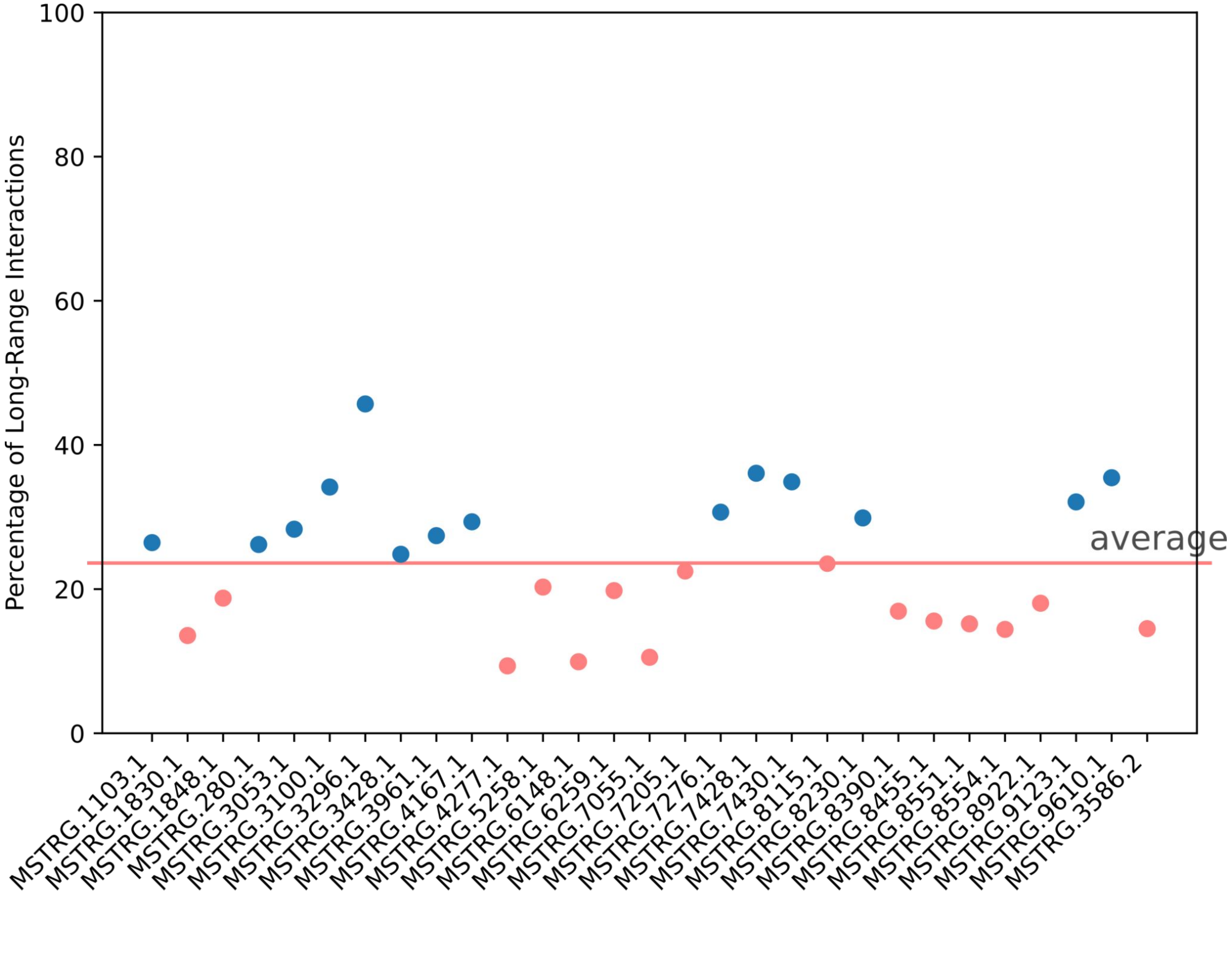

Figure S7. Visual representation of the distribution of long-range interactions across the analyzed lncRNA sequences. The x-axis of the plot corresponds to the unique identifier of each lncRNA, while the y-axis represents the calculated percentage of long-range interactions. The red line represents the dataset's mean percentage of long-range interactions, which is 22.6%. Data points in red indicate lncRNAs that are below this average.

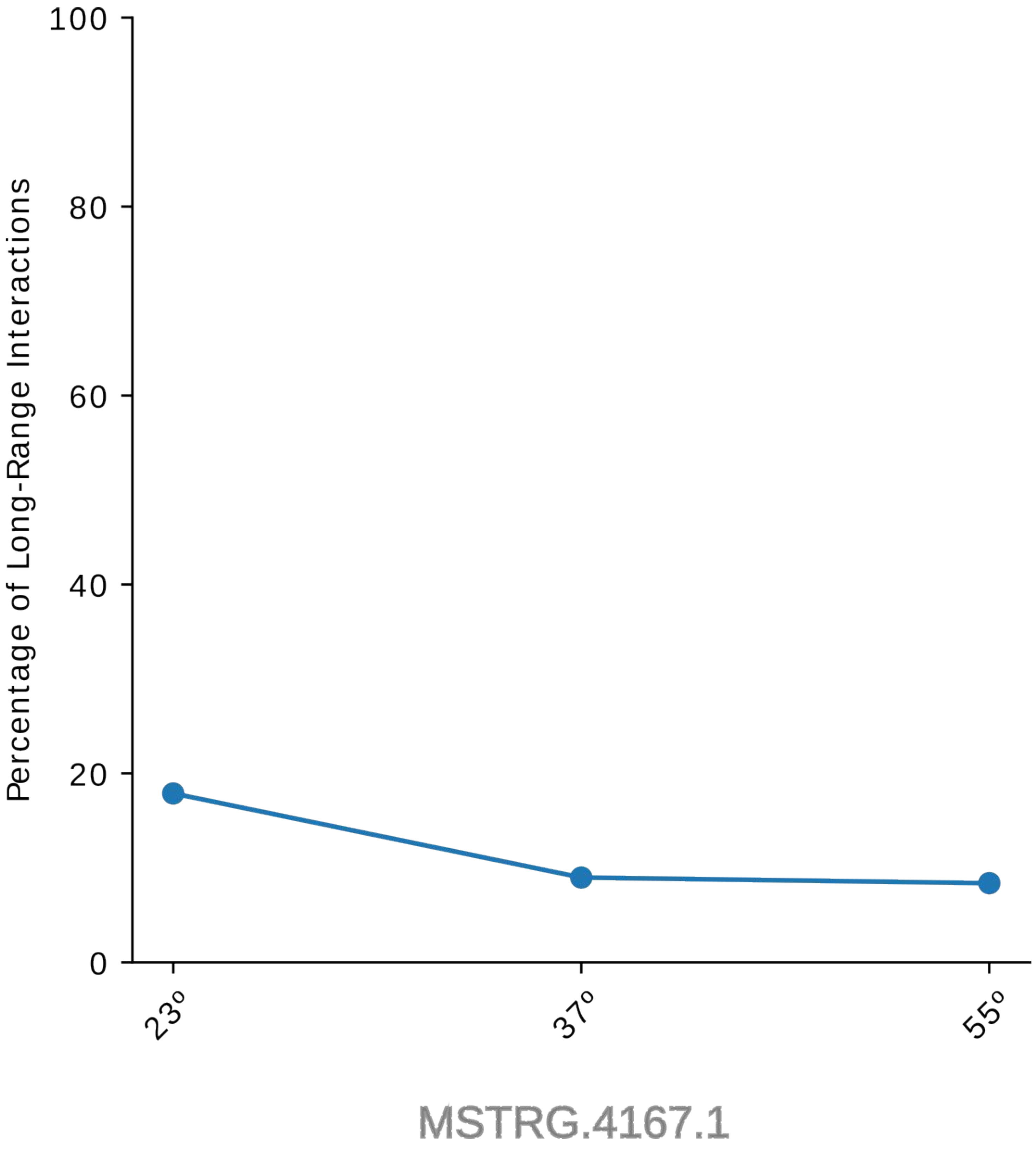

Figure S8. Percentage of long-range interactions of MSTRG.4167.1 lncRNAs at three different temperatures

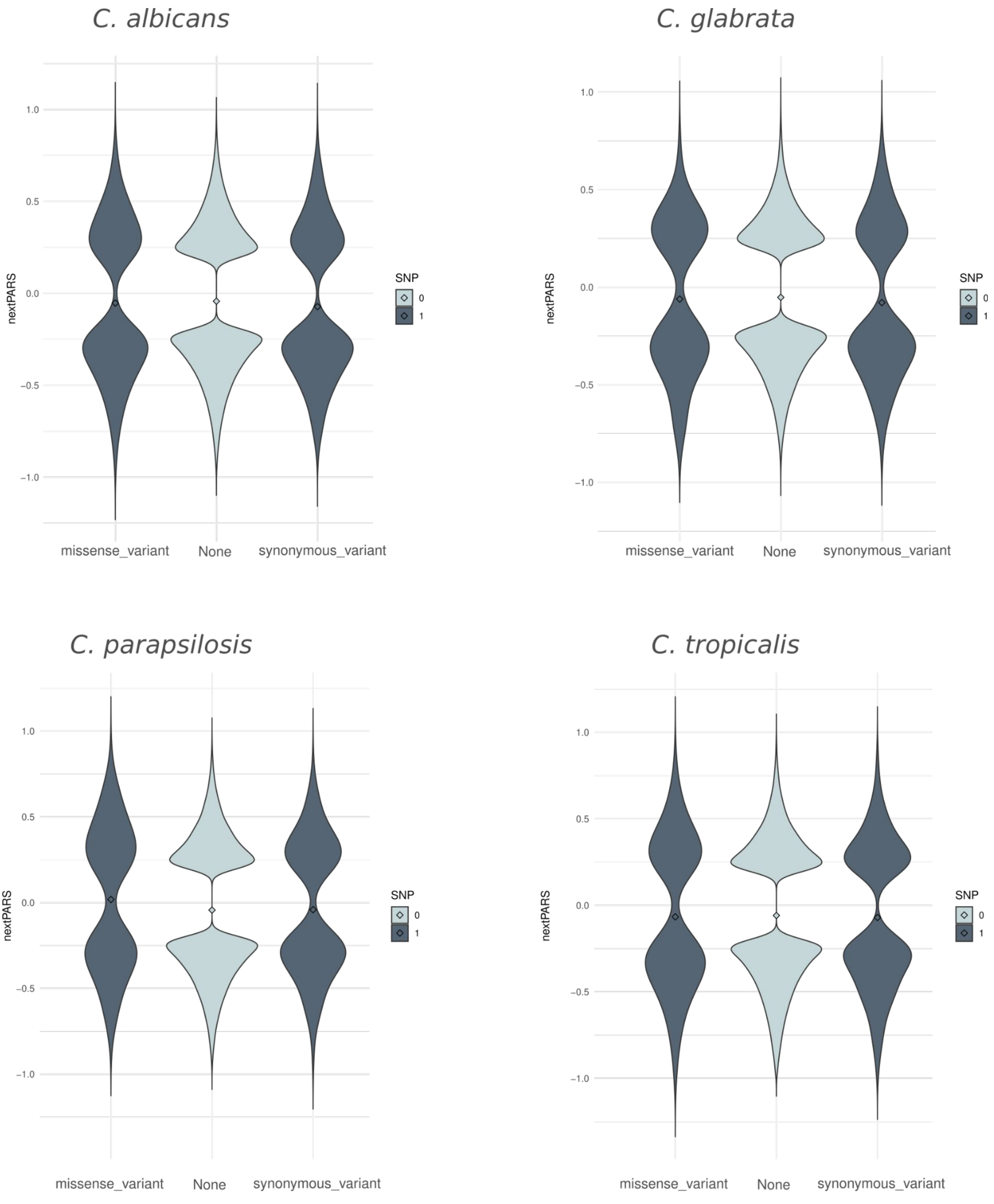

Figure S9. Violin plot comparing nextPARS scores in genomic loci with single nucleotide polymorphisms (SNPs) to those without reported SNPs across four Candida species. SNP positions leading to amino acid changes (missense_variant) and those that are silent (synonymous_variant) are shown as separate groups.

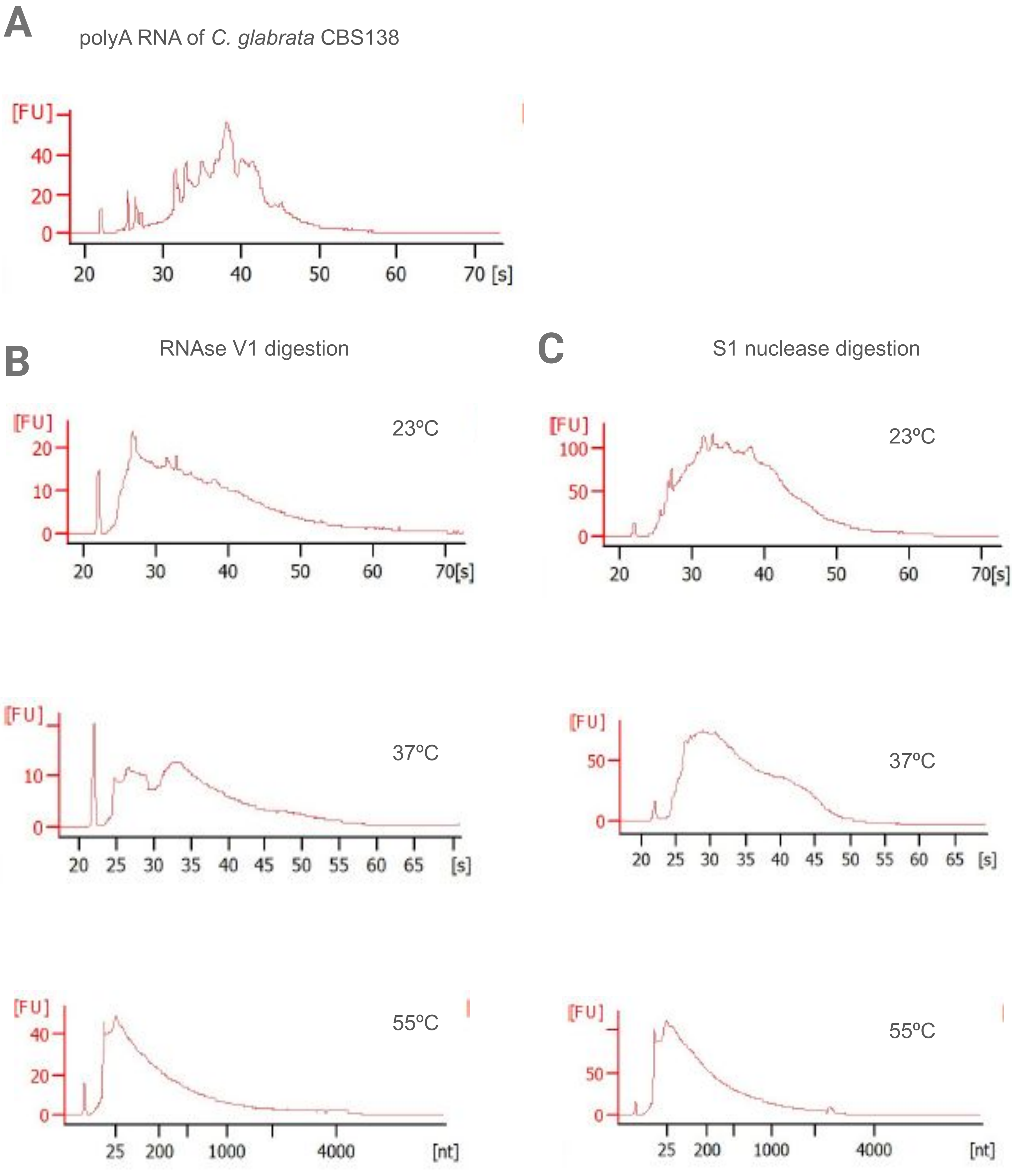

Figure S10. Agilent 2100 Bioanalyzer profiles of RNA samples used in the experiments. The profiles show the arbitrary fluorescence units (FU) after subtracting local background signal from total signal in the y axis, and size of the analyzed molecules in time scale (seconds) or nucleotides (nt) in the x axis. **(A)** Profile of polyA RNA from C.glabrata CBS138. **(B)** Profiles of the polyA RNA after having been folded and digested with RNase V1 at 23ºC, 37ºC or 55ºC. **(C)** Profiles of the polyA RNA after having been folded and digested with S1 nuclease at 23ºC, 37ºC or 55ºC.

	C. albicans	*C. glabrata*	*C. parapsilosis*	*C. tropicalis*
**PCG**	286	296	412	285
**lncRNAs**	5	1	3	19

Table S1. Number of mRNAs and lncRNAs detected with sufficient confidence (> five average counts per position) in the four *Candida* species

MSTRG.4167.1	23º vs 37º	37º vs º55	23º vs 55º
**Similarity score**	6.634375	6.164139	5.88634

Table S2. Similarity scores for the structural comparison of lncRNA MSTRG.4167.1 at different temperatures (23°C, 37°C, and 55°C).
